# Unique Eomes^+^ NK Cell Subsets Are Present in Uterus and Decidua During Early Pregnancy

**DOI:** 10.3389/fimmu.2015.00646

**Published:** 2016-01-07

**Authors:** Elisa Montaldo, Paola Vacca, Laura Chiossone, Daniele Croxatto, Fabrizio Loiacono, Stefania Martini, Simone Ferrero, Thierry Walzer, Lorenzo Moretta, Maria Cristina Mingari

**Affiliations:** ^1^G. Gaslini Institute, Genoa, Italy; ^2^Department of Experimental Medicine (DIMES), Università degli Studi di Genova, Genoa, Italy; ^3^IRCCS AOU San Martino-IST, Genoa, Italy; ^4^Department of Neurosciences Rehabilitation Ophthalmology, Genetics, Maternal and Child Health (DiNOGMI), Università degli Studi di Genova, Genoa, Italy; ^5^CIRI, Centre International de Recherche en Infectiologie – INSERM, Ecole Normale Supérieure de Lyon, Université Lyon 1, CNRS, Lyon, France; ^6^Department of Immunology, IRCCS Bambino Gesù Children’s Hospital, Rome, Italy

**Keywords:** ILC, NK cells, ILC1, Eomes, pregnancy, tissue-resident NK cells

## Abstract

Decidual and uterine natural killer (NK) cells have been shown to contribute to the successful pregnancy both in humans and mice. NK cells represent “cytotoxic” group 1 innate lymphoid cells (ILCs) and are distinct from the recently described “helper” ILC1. Here, we show that both in humans and mice the majority of group 1 ILC in endometrium/uterus and decidua express Eomesodermin (Eomes), thus suggesting that they are developmentally related to conventional NK cells. However, they differ from peripheral NK cells. In humans, Eomes^+^ decidual NK (dNK) cells express CD49a and other markers of tissue residency, including CD103, integrin β7, CD9, and CD69. The expression of CD103 allows the identification of different subsets of IFNγ-producing Eomes^+^ NK cells. We show that TGFβ can sustain/induce CD103 and CD9 expression in dNK cells and decidual CD34-derived NK cells, indicating that the decidual microenvironment can instruct the phenotype of Eomes^+^ NK cells. In murine decidua and uterus, Eomes^+^ cells include CD49a^−^CD49b^+^ conventional NK cells and CD49a^+^ cells. Notably, Eomes^+^CD49a^+^ cells are absent in spleen and liver. Decidual and uterine Eomes^+^CD49a^+^ cells can be dissected in two peculiar cell subsets according to CD49b expression. CD49a^+^CD49b^^−^^ and CD49a^+^CD49b^+^ cells are enriched in immature CD11b^low^CD27^high^ cells, while CD49a^−^CD49b^+^ cells contain higher percentages of mature CD11b^high^CD27^low^ cells, both in uterus and decidua. Moreover, Eomes^+^CD49a^+^CD49b^−^ cells decrease during gestation, thus suggesting that this peculiar subset may be required in early pregnancy rather than on later phases. Conversely, a minor Eomes^−^CD49a^+^ ILC1 population present in decidua and uterus increases during pregnancy. CD49b^−^Eomes^±^ cells produce mainly TNF, while CD49a^−^CD49b^+^ conventional NK cells and CD49a^+^CD49b^+^ cells produce both IFNγ and TNF. Thus, human and murine decidua contains unique subsets of group 1 ILCs, including Eomes^+^ and Eomes^−^ cells, with peculiar phenotypic and functional features. Our study contributes to re-examination of the complexity of uterine and decidual ILC subsets in humans and mice and highlights the role of the decidual microenvironment in shaping the features of these cells.

## Introduction

Innate lymphoid cells (ILCs) represent a family of lymphocytes that differ from B and T cells since they lack recombination activating gene (RAG)-dependent rearranged antigen receptors. ILCs share the dependence on Id2 transcriptional repressor and on the common γ chain cytokine receptor for their development. ILCs have been classified into three main groups according to their transcription factor and cytokine profile. Group 1 ILCs express the T-box transcription factor T-bet (*Tbx21*) and mainly express IFNγ and TNF. Group 2 ILCs (ILC2) depend on GATA binding protein-3 transcription factor and produce type-2 cytokines. Finally, group 3 ILCs (ILC3) express the retinoic acid receptor (RAR)-related orphan receptor (ROR)γt, and produce IL-17 and IL-22 (1, 2).

Group 1 ILCs include “helper-ILC1” (hereinafter referred to as ILC1) and “cytotoxic-ILCs,” i.e., natural killer (NK) cells (2). ILC1 mainly express IFNγ and TNF and provide defenses against intracellular bacteria and protozoa. Conversely, NK cells, beside IFNγ production, also display cytolytic activity against virus-infected or tumor cells. Unlike ILC1, NK cells also express the transcription factor Eomesodermin (Eomes) (3). ILC1 appear to be resident populations in intestine, liver, and uterus, while NK cells are present in tissues and recirculate in the blood (4–9). A peculiar subset of NK cells residing in murine liver has been recently described and termed tissue-resident NK (trNK) cells (10). These cells express T-bet, but not Eomes. Liver trNK cells display striking phenotypical similarities with ILC1 described in mucosal tissues (11, 12), suggesting a partial overlap between these two cell subsets. Indeed, studies regarding ILC lineage specifications suggested that liver trNK cells are more related to ILC1 than to “conventional” splenic NK (cNK) cells (5). Moreover, Daussy et al. demonstrated that liver NK1.1^+^T-bet^+^Eomes^+^ and NK1.1^+^T-bet^+^Eomes^−^ cells represent two distinct lineages of differentiation, which derive from precursors of medullary and peripheral origin, respectively (6, 9). Of note, peculiar Eomes^+^ NK cells, differing from cNK and ILC1, have been identified in murine salivary glands and uterus (7, 13).

Innate immune cells are important components of decidual microenvironment during pregnancy (14–16). Among ILCs, we recently identified ILC3 in human decidua during early pregnancy (17). However, the best-characterized and more abundant ILC population is that of NK cells that, during the first trimester of pregnancy, represents up to 70% of decidual infiltrating lymphocytes (DILs). Human decidual NK (dNK) cells are characterized by CD56^bright^CD16^−^KIR^+^CD9^+^CD49a^+^ phenotype, are poorly cytolytic, and produce low amounts of IFNγ, as compared to peripheral blood (PB) NK cells (15, 18–20). On the other hand, dNK cells secrete cytokines and chemokines that promote neo-angiogenesis, tissue remodeling and placentation (16). Similar to humans, also murine dNK cells are abundant during the early phase of pregnancy and display unique phenotypic and functional features (21). Since we have previously shown that uterine (u)NK and dNK cells may originate, at least in part, from *in situ* precursors (21) and in light of recent evidences about ILC complexity and differentiation (6, 9, 10), here we re-evaluated the nature of uterine and dNK cells in humans and mice, in order to clarify whether they may be ascribed to ILCs previously identified in other tissues or rather represent unique subsets only present in uterus and decidua.

## Materials and Methods

### Isolation of Human Cells

Endometrial biopsies were obtained from normally cycling women undergoing surgery for ovarian cyst removal at IRCCS AOU San Martino-IST (Genova, Italy). Decidua (d) samples were obtained at 9–12 weeks of gestation from singleton pregnancies of mothers requesting termination of pregnancy for social reasons at IRCCS AOU San Martino-IST (Genova, Italy). The relevant institutional review boards approved the study and all patients gave their written informed consent according to the Declaration of Helsinki. We isolated cell suspensions from decidual and endometrial tissue with GentleMacs (Miltenyi Biotec, Bergisch Gladbach, Germany) and cells were then filtered as previously described (22). Decidua and endometrial infiltrating lymphocytes were isolated by Fycoll (Cedarlane, Burlington, ON, Canada) gradient centrifugation. Lymphocytes obtained were subsequently analyzed by flow cytometry, stimulated for cytokine production, or sorted for subsequent stimulation and culture. In order to isolate ILC subsets and CD34^+^ cells, DILs were sorted as (CD45^+^CD3^−^CD14^−^CD34^−^CD56^+^CD127^−^CD117^−^)-NKp44^+^CD103^+^, -NKp44^−^CD103^+^, and -NKp44^−^CD103^−^ cells and CD45^+^CD14^−^CD19^−^CD3^−^CD56^−^CD34^+^ cells at FACSAria (BD Bioscience, San Jose, CA, USA), purity was routinely >95%. Decidual stromal cells (dSC) were isolated as previously described (23). PB NK cells were isolated with Human NK cell enrichment cocktail-RosetteSep (StemCell technologies).

### ILC Culture, Analysis of Cytokine Production, and Degranulation

Innate lymphoid cell subsets were cultured in U-bottom 96-well plates (Corning, Tewksbury, MA, USA) in RPMI-1640 medium (Lonza, Basel, Switzerland) supplemented with 10% (vol/vol) FCS (Lonza), 1% (vol/vol) glutamine, and 1% (vol/vol) penicillin, neomycin, and streptomycin antibiotic mixture (Lonza and Cambrex, Charles City, IA, USA). When indicated we added 10 ng/ml IL-15 (Miltenyi) and 5 ng/ml recombinant TGFβ (Peprotech). To perform co-culture experiments, dSC and NK cells were plated at the ratio of 1:5 ± α-TGFβ neutralizing antibody (R&D). CD34^+^ cells were cultured in the presence of RPMI-1640 medium supplemented with 10% (vol/vol) human AB serum (Lonza), 1% (vol/vol) glutamine, and 1% (vol/vol) penicillin, neomycin, and streptomycin antibiotic mixture in the presence of 10 ng/ml Flt3-L, 20 ng/ml SCF, IL-7, IL-15, and IL-21 (Milteny) ±5 ng/ml TGFβ. For the analysis of cytokine production, cells were stimulated as indicated in figures with 25 ng/ml PMA, 1 μg/ml Ionomycin (Sigma-Aldrich), 50 ng/ml IL-23, 50 ng/ml IL-15, 10 ng/ml IL-12 (Miltenyi), and 100 ng/ml IL-18 (MBL). To perform intracellular cytokine analysis, cells were stimulated 18 h in the presence of Brefeldin A (BD Bioscience). After stimulation, cells were stained for surface markers, fixed with Cytofix/Cytoperm, and permeabilized with Perm/Wash (BD Bioscience) according to the manufacturer’s instructions. To perform supernatants (spt) cell analysis, ILCs were stimulated for 72 h, the spt were collected, and cytokine concentration was evaluated by ELISA multiplex assay (Merck Millipore) and analyzed with Magpix system (Luminex). TGFβ produced by dSC was measured by ELISA multiplex assay in spt collected after 1 week of culture in serum-supplemented RPMI-1640 medium. To perform degranulation assays coupled with analysis of IFNγ production, 72-h-cytokine-activated DILs were co-cultured with K562 cells at an effector:target (E:T) ratio of 1:1, in the presence of anti-CD107a and Monensin (BD Bioscience). After 4 h, cells were washed and stained for surface and intracellular markers.

### Mice, Collection of Decidual and Uterine Tissues, and Cell Isolation

C57BL/6 mice were purchased from Charles River (Como, Italy). Eomes-GFP reporter mice (6) were maintained and mated at the Animal Facility of the IRCCS-AOU San Martino-IST. All mice were used between 6 and 12 weeks of age. Housing and treatments of animals were in accordance with the Italian and European Community guidelines (D.L. 2711/92 No.116; 86/609/EEC Directive) and approved by the internal Ethic Committee. To time pregnant females, superovulation was induced by intraperitoneal injection of 5 IU of Pregnant Mares Serum (Folligon; Intervet, Italy) followed, 48 h later, by 5 additional IU of hCG (Corulon; Intervet, Italy). Immediately following injection, each female was mated with a syngeneic male overnight. Females with copulation plug were separated and identified as gestation day (gd) 0.5. Mice were killed at different gd by cervical dislocation and uterus was processed as previously described (21). Lymphoid cells present in the implant before gd 9 are of maternal origin since fetal hematopoiesis starts at gd 9. Thus, at gd 5.5 uterus was open and the implants were isolated and processed as a source of decidual tissue; while at gd 10.5 and 14.5, the decidua was separated from the implant by cutting away the mesometrial pole and all decidua derived from the same uterus were pooled. Uterine wall, once cleared out of the implants, was further processed. The uteri of virgin females were isolated and processed entirely. Decidual and uterine tissues were mechanically disrupted. Spleen and liver were also collected from virgin and pregnant mice and single-cell suspensions were prepared as previously described (24).

### Flow Cytometry Analyses and Monoclonal Antibodies

Human and mouse cells were stained with the monoclonal antibodies listed in Table 1. Before staining with mAbs, murine cells were incubated with FcR blocking reagent (Miltenyi). For intranuclear staining of transcription factor, cells were stained for surface markers, fixed with Fixation/Permeabilization buffer and permeabilized with permeabilization buffer (eBioscience), respectively, according to the manufacturer’s instructions. All samples were analyzed on Gallios Flow Cytometer (Beckman Coulter) or MACSQuant Analyzer (Miltenyi). Data were analyzed with FlowJo software (TreeStar, Ashland, OR, USA). Unstained cells were used as negative controls and markers set accordingly.

**Table 1:** **List of reagents used for flow cytometry analysis**.

Marker	Fluorocrome	Supplier	Reactivity
β7	APC	BD Bioscience	h/m
CD3	ECD	Beckman Coulter	h
CD3	PE	Miltenyi	h
CD3	PE/Dazzle594	BioLegend	m
CD3	PacificBlue	BioLegend	m
CD9	PE	Miltenyi	h
CD9	PE	eBioscience	m
CD14	APC-eFluor480	eBioscience	h
CD14	ECD	Beckman Coulter	h
CD34	FITC	Miltenyi	h
CD45	APC-H7	BD Bioscience	h
CD45	biotin	BD Bioscience	m
CD49a	APC-Vio770	Miltenyi	h
CD49a	APC	BioLegend	m
CD49b	PacificBlue	BioLegend	m
CD49b	PE	Miltenyi	m
CD56	PC7	Beckman Coulter	h
CD69	PE	Miltenyi	h
CD69	biotin	eBioscience	m
CD94	FITC	BioLegend	h
CD103	PE	BioLegend	h
CD103	FITC	BioLegend	h
CD107a	FITC	BD Bioscience	h
CD117	APC	Miltenyi	h
CD117	PerCP-Cy5.5	BioLegend	h
CD122	PE	BD Bioscience	m
CD122	biotin	BD Bioscience	m
CD127	BrilliantViolet421	BioLegend	h
CD127	PerCP-Cy5.5	BioLegend	h
CD158a,h	PE	Beckman Coulter	h
CD158b1/b2,j	PE	Beckman Coulter	h
CD158e1,e2	PE	Beckman Coulter	h
CD160	eFluor660	eBioscience	m
CD314 (NKG2D)	PE	Miltenyi	h
CD335 (NKp46)	APC	Miltenyi	h
CD336 (NKp44)	APC	BioLegend	h
CD337 (NKp30)	PE	Miltenyi	h
NKp46	PE	eBioscience	m
NKp46	eFluor660	eBioscience	m
Eomes	AlexaFluor647	eBioscience	h
GranzymeA	PE	BD Bioscience	h
GranzymeB	PE	Life Technologies	h
IFNγ	Alexa647	BD Bioscience	h
IFNγ	PerCP-Cy5.5	eBioscience	h
IFNγ	PECy7	BD Bioscience	m
IL-22	PE	eBioscience	h
NK1.1	APC	BioLegend	m
NK1.1	PerCP-Cy5.5	eBioscience	m
Perforin	PE	Ancell	h
RORγt	PE	eBioscience	h/m
TNFα	eFluor450	eBioscience	h
TNF	PE	Miltenyi	m
TRAIL	VioBlue	Miltenyi	m
Live/dead fixable	Aqua Dead	Life Technologies	
Streptavidin	Alexa-fluor700	Life Technologies	
Streptavidin	PE	Life Technologies	
Streptavidin	eFluor710	eBioscience	

### Statistical Analysis

Prism6 GraphPad software was used for statistical analysis. Figures 2C,D, 4A,C and 5B show one-way ANOVA. Figures 4E– G and 5H show two-way ANOVA. Figure 5F show one-way ANOVA plus post test for linear trend. We considered significant *p*-values ≤0.05.

## Results

### Distinct Eomes^+^ NK Subsets Are Present in Human Endometrium and Decidua

**Figure 1:**
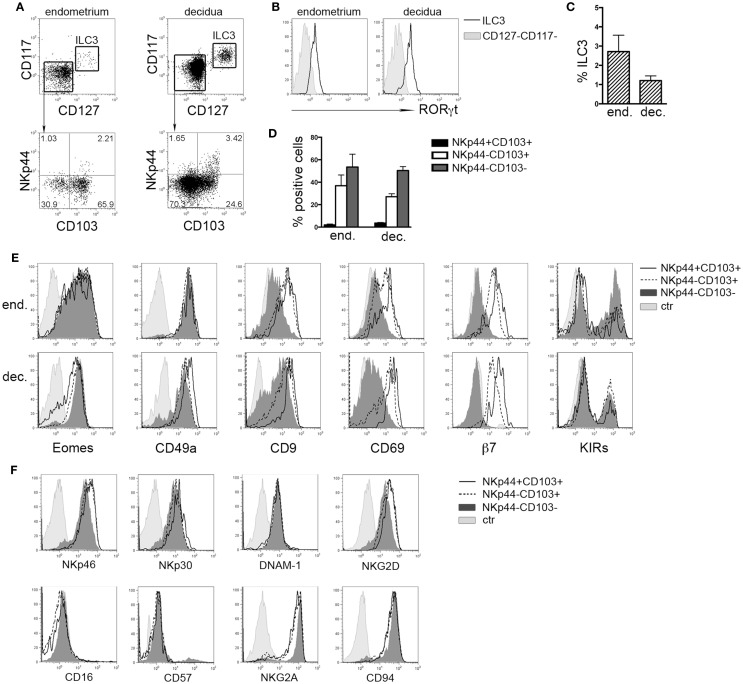
***Ex vivo* characterization of human endometrial and decidual ILC subsets**. **(A)** Identification of different ILC subsets by surface marker analysis, after gating on Lin^−^CD56^+^ cells. **(B)** Intranuclear expression of RORγt (*n* = 4–8). **(C)** Mean ± SEM of Lin^−^CD127^+^CD117^+^ ILC3 (*n* = 7). **(D)** Percentage of different NK cell subsets after gating on endometrial and decidual Lin^−^CD56^+^CD127^−^CD117^−^ cells (mean ± SEM; *n* = 6 and 28, respectively). **(E,F)** Phenotypic analysis of endometrial **(E)** and decidual **(E,F)** NKp44^+^CD103^+^, NKp44^−^CD103^+^, and NKp44^−^CD103^−^ cells. Control (ctr) corresponds to unstained cells. One representative experiment out of 8 performed.

It has been shown that in tonsil and gastrointestinal epithelium NKp44 molecule is expressed by CD56^+^CD127^+^RORγt^+^ ILC3 and CD56^+^CD127^−^CD103^+^ ILCs (8). Lymphoid cells isolated from human endometrium and decidua contained similar percentages of Lin^−^CD56^+^CD127^+^CD117^+^ RORγt^+^ ILC3 (Figures 1A–C) that homogeneously expressed NKp44 (not shown) (7, 17). Among Lin^−^CD56^+^CD127^−^CD117^−^RORγt^−^ cells, we identified three subsets according to NKp44 and CD103 surface expression (Figures 1A,D). In particular, NKp44^+^CD103^+^ subset represented a minor fraction of CD56^+^ cells as compared to NKp44^−^CD103^+^ and NKp44^−^CD103^−^ cells (Figures 1A,D). The frequency of these three cell subsets did not significantly differ between endometrium and decidua. Thus, the presence of these ILC subsets seems not to depend on pregnancy status. NKp44^+^CD103^+^, NKp44^−^CD103^+^, and NKp44^−^CD103^−^ cells expressed T-bet and Eomes (Figure 1E and not shown), thus strongly suggesting that they belong to the NK cell lineage. The analysis of markers commonly used to identify endometrial and dNK cells (15) revealed that all three subsets were CD49a^+^, while CD9 and CD69 were expressed at higher levels by NKp44^+^CD103^+^ and NKp44^−^CD103^+^ cells than by NKp44^−^CD103^−^ cells. In addition, CD103^+^ subsets expressed integrin β_7_ that, together with CD103, can forms the α_E_β_7_ heterodimer. Moreover, the main activating NK cell receptors, including NKp46, NKp30, NKG2D, and DNAM-1 were homogenously expressed by all three cell populations (Figure 1F). All subsets were CD16^−^ CD57^−^and CD94/NKG2A^+^ (Figure 1F), while expressing variable amounts of KIRs (Figure 1E).

### Decidual Stromal Cells-Derived TGFβ Influences dNK Cell Phenotype

**Figure 2:**
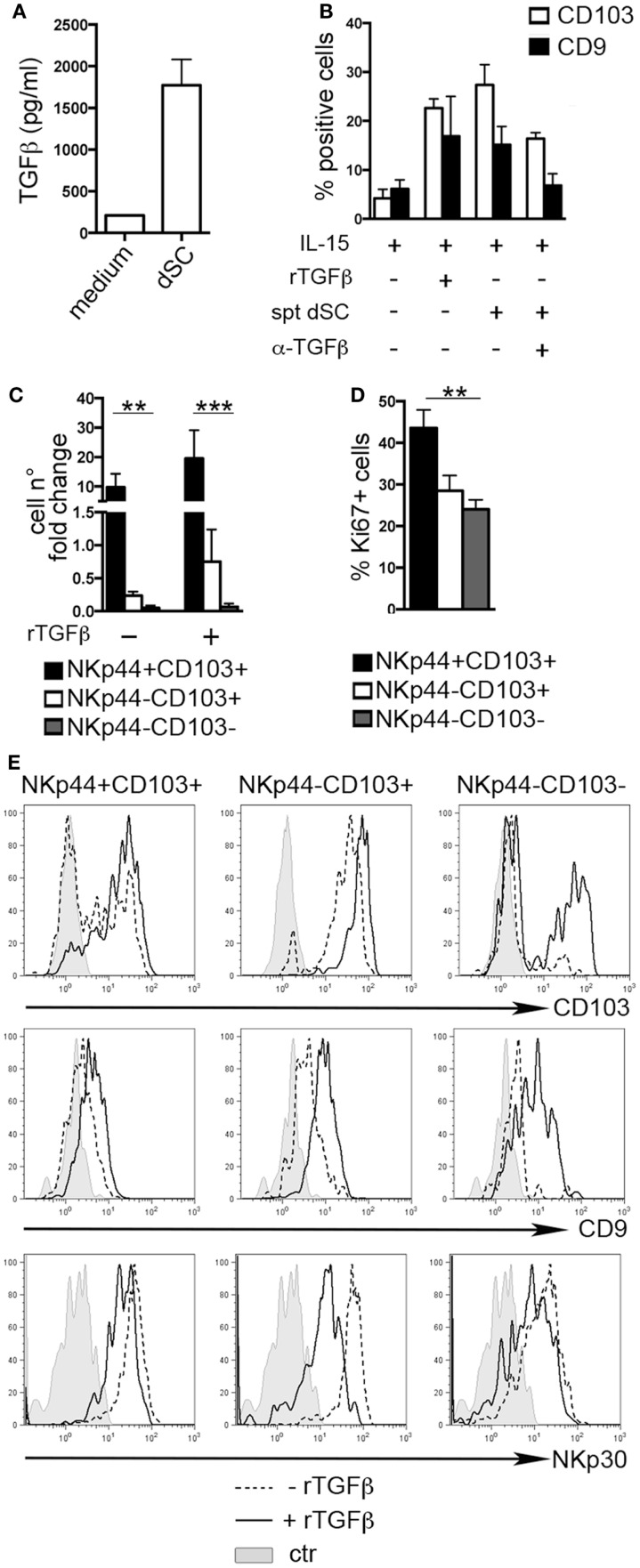
**TGFβ modulates human dNK cell phenotype**. **(A)** TGFβ concentration (picogram/milliliter) in dSC spt derived from 8 different donors. **(B)** PB NK cells were cultured as indicated for 15 days and analyzed by flow cytometry for the expression of CD103 and CD9; mean ± SEM of positive cells (*n* = 5). **(C)** NKp44^+^CD103^+^, NKp44^−^CD103^+^, and NKp44^−^CD103^−^ dNK cell subsets were sorted and cultured with IL-15 ± rTGFβ. Cell number fold change is depicted, calculated as ratio of cell number at day 20 to that at day 0. **(D)** Ki67 expression on DILs after gating on dNK cell subsets (*n* = 6). **(E)** Sorted dNK cell subsets were cultured for 20 days in IL-15 ± TGFβ and analyzed for the expression of the indicated markers, one representative experiment out of 3 performed. Control (ctr) corresponds to unstained cells.

The typical features of dNK cells depend, at least in part, from the influence of decidual microenvironment. Previous reports indicated CD103 and CD9 as markers of exposure to TGFβ (8, 25, 26). Endometrial and dSC were shown to produce molecules of the TGFβ family (27). Accordingly, we found that dSC isolated from different donors produced TGFβ (Figure 2A). dSC-derived spt or recombinant (r) TGFβ induced *de novo* expression of CD103 and CD9 on PB NK cells. In addition, in the presence of anti-TGFβ (α-TGFβ) neutralizing antibody, the expression of both markers was inhibited (Figure 2B). Next, we evaluated the effect of rTGFβ on the three dNK subsets identified. Only the NKp44^+^CD103^+^ subset underwent *in vitro* cell proliferation upon 7 days culture (Figure 2C). This result was in accordance with higher *ex vivo* expression of Ki67 (Figure 2D). Although TGFβ did not influence cell proliferation (Figure 2C), it did affect the phenotypic features of the three subsets. In particular, NKp44^+^CD103^+^ retained CD103 expression only when cultured in the presence of rTGFβ (Figure 2E). In agreement with previous studies, NKp44^−^CD103^−^ cells cultured with rTGFβ acquired CD103 (8, 26). CD9 expression was partially downregulated when cells were cultured in the absence of TGFβ (Figure 2E). In addition, rTGFβ reduced the expression of NKp30, particularly in NKp44^−^CD103^+^ cells (Figure 2E) (28).

**Figure 3:**
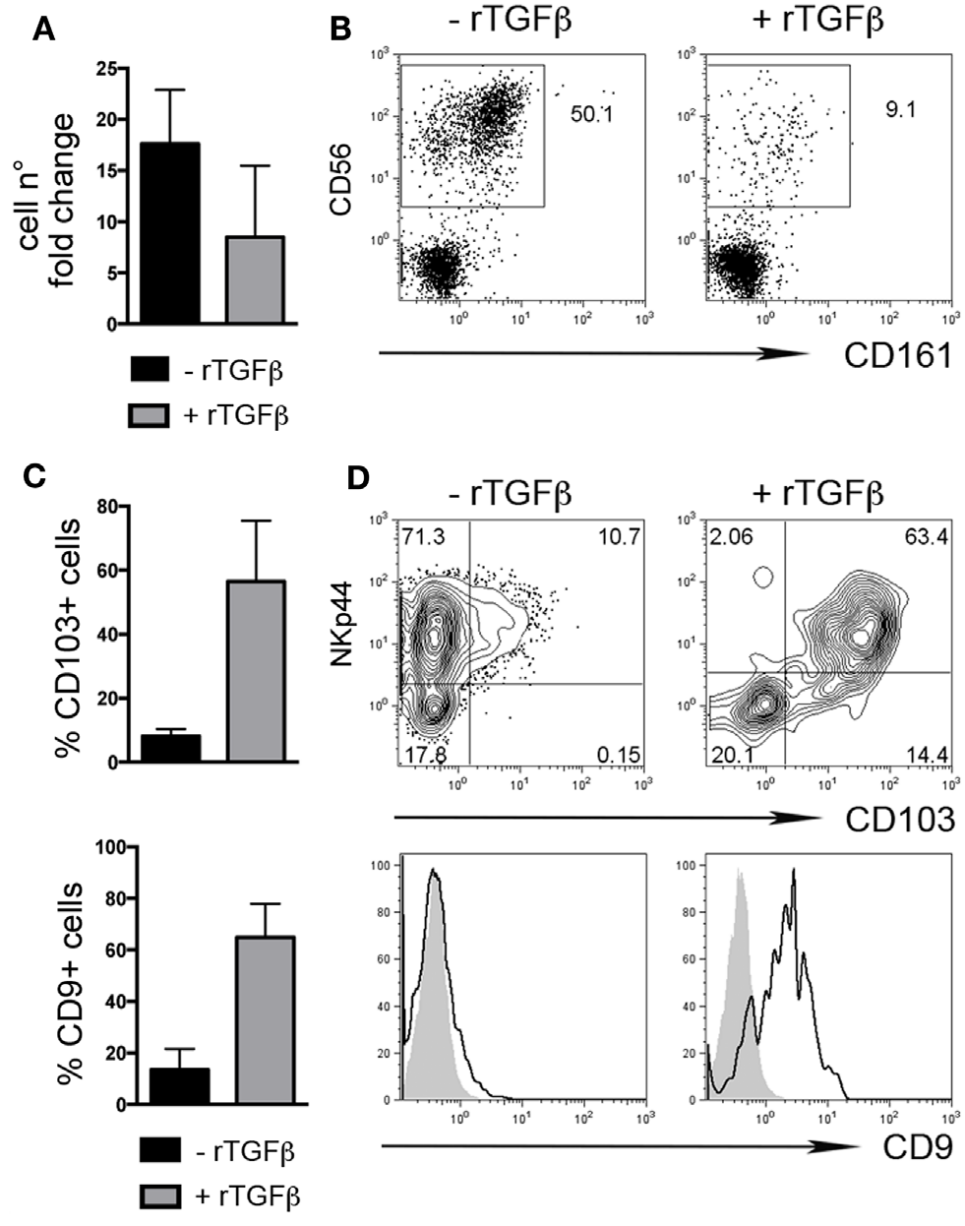
**TGFβ modulates human dCD34^+^ cell differentiation toward CD56^+^CD103^+^CD9^+^ cells**. Purified dCD34^+^ cells were cultured 14 days in the presence of Flt3-L, SCF, IL-7, IL-15, and IL-21 ± rTGFβ **(A)** Cell number fold change calculated as ratio of cell number at day 14 to that at day 0. **(B)** Flow cytometry analysis of *in vitro* differentiated cells after gating on Lin^−^ cells. **(C,D)** Flow cytometry analysis of *in vitro* differentiated cells after gating on Lin^−^CD56^+^ cells, **(C)** mean ± SEM of positive cells (*n* = 3), and **(D)** one representative experiment, gray histogram corresponds to unstained cells, black line gated on Lin^−^CD56^+^ cells.

We also investigated whether rTGFβ could influence dNK cell differentiation from dCD34^+^ hematopoietic precursors. In the presence of rTGFβ, dCD34^+^ cell number fold expansion was affected in two out of three experiments (Figure 3A). Differentiation toward Lin-CD56^+^CD161^±^ cells was inhibited (Figure 3B). However, CD56^+^ cells expressed higher levels of CD103 and CD9 than cells cultured in the absence of rTGFβ (Figures 3C,D). Thus, it is conceivable that uterine/decidual microenvironment, enriched in TGFβ, may play a relevant role in the induction of unique features in recruited or *in situ* differentiated NK cells and in the maintenance of dNK cell phenotype.

### CD103^+^ Cells Represent the Major Source of IFNγ in dNK Cells

**Figure 4:**
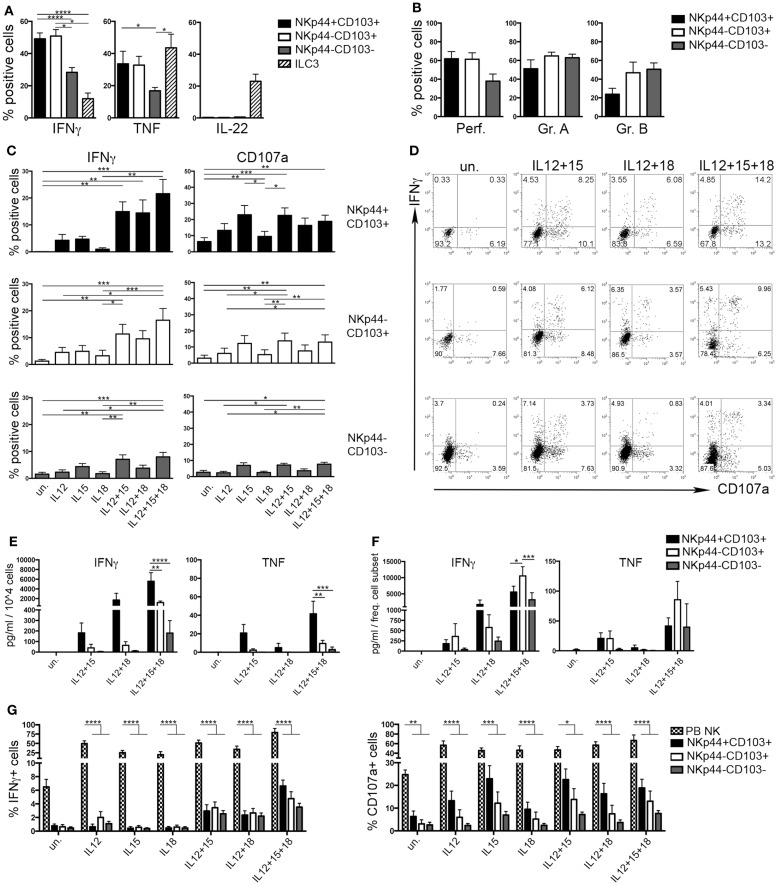
**Human decidual NKp44^+^CD103^+^, NKp44^−^CD103^+^, and NKp44^−^CD103^−^ cells produce IFNγ upon cytokine stimulation**. **(A)** DILs were stimulated with P + I + IL-23 for 18 h and analyzed for intracellular cytokine expression after gating on the three dNK cell subsets and ILC3. Mean ± SEM of cytokine positive cells (*n* = 11). **(B)** DILs were analyzed for the intracellular expression of perforin and granzymes. Mean ± SEM of positive cells (*n* = 6). **(C,D)** DILs were stimulated with IL-12, IL-15, and IL-18 alone or in combination. After 72 h, cells were incubated 4 h with K562 cells and analyzed for the expression of IFNγ and CD107a. **(C)** Mean ± SEM of positive cells (*n* = 6) and **(D)** one representative experiment. **(E,F)** dNK cell subsets were sorted and stimulated as indicated. Cell spt were collected after 72 h and analyzed by ELISA multiplex assay for IFNγ and TNF. For statistical analysis, within each stimulation condition, data referred to different cell populations were compared with Tukey’s multiple comparison. **(E)** Mean ± SEM of cytokine concentration (picogram/milliliter) produced by 10^4^ cells (*n* = 5). **(F)** Mean ± SEM of cytokine concentration (picogram/milliliter) is normalized to the mean frequency (see Figure 1D) of each subset (*n* = 5). **(G)** DILs and PB NK cells were stimulated as indicated. After 72 h, cells were incubated 4 h with K562 cells and analyzed for the expression of IFNγ and CD107a; mean ± SEM of positive cells (*n* = 6).

During the early phases of pregnancy, the balance between inflammation and tolerance is critical (29). A successful pregnancy needs a “regulatory phase” that inhibits immuno-mediated fetal rejection. However, an early “inflammatory phase” favors embryo implantation thanks to the production of cytokines and chemokines that contribute to tissue remodeling and neo-angiogenesis (30). Analysis of cytokines produced by the three dNK subsets revealed that CD103^+^ cells expressed higher amounts of IFNγ and TNF than CD103^−^ cells upon stimulation (Figure 4A). IL-22 was exclusively produced by ILC3 (Figure 4A). dNK cells are classically considered as poorly cytotoxic, in spite of their content of cytolytic granules (15). All dNK subsets expressed similar levels of perforin, granzymes A and B (Figure 4B). After 18 h of stimulation with different cytokine combinations, dNK cell subsets were co-cultured with K562 target cells and analyzed for IFNγ production and CD107a expression. Stimulation of dNK subsets with IL-12 or IL-15, or IL-18 did not induce significant IFNγ production (Figures 4C,D). Conversely, cells stimulated with IL-12 + IL-15 expressed higher amounts of IFNγ than unstimulated cells. Moreover, cells cultured with IL-12 + IL-15 + IL-18 produced the highest amounts of IFNγ (Figures 4C–E), highlighting the known synergy between these cytokines (31). CD107a expression was enhanced upon cell stimulation with IL-15, either alone or in combination with other cytokines (Figures 4C,D). Remarkably, although NKp44^+^CD103^+^ cells produced the highest amounts of IFNγ and TNF on a per cell basis (Figure 4E), this cell subset represented only 2% of CD56^+^CD127^−^CD117^−^ cells (see Figure 1D). Indeed, when the amount of cytokines produced was normalized to the relative frequency of the cell subsets, NKp44^−^CD103^+^ cells resulted as the most important source of IFNγ (Figure 4F). In any case, all three dNK subsets displayed both lower IFNγ production and CD107a expression than PB NK cells (Figure 4G), in line with previous reports (23, 26, 32). Thus, decidual microenvironment is likely to affect both the phenotypical and the functional features typical of NK cells.

### Murine Uterus and Decidua Contain Eomes^+^CD49a^+^CD49b^+^ and Eomes^+^CD49a^+^CD49b^−^ NK Cell Subsets

**Figure 5:**
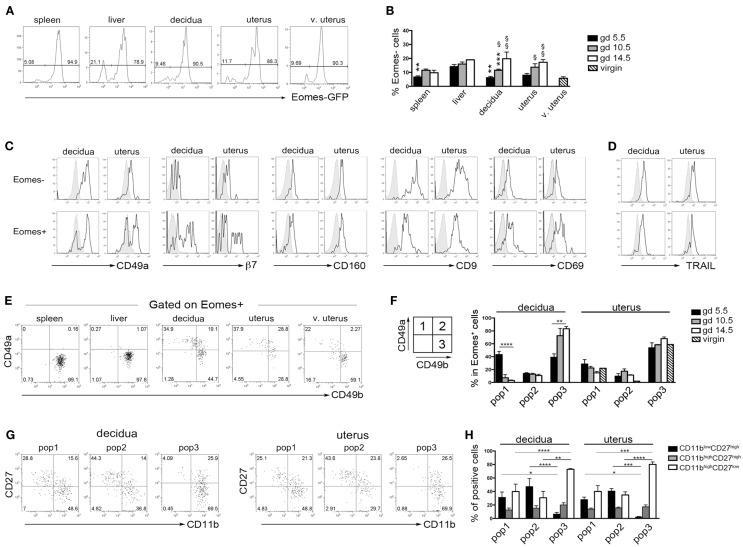
**Peculiar subsets of Eomes^+^ decidual and uterine murine NK cells during pregnancy**. **(A)** Analysis of Eomes expression in CD3^−^NK1.1^+^ cells in the indicated organs isolated from Eomes-GFP mice at gd 5.5; one representative experiment (*n* = 25). **(B)** Mean ± SEM of Eomes^−^ cell percentages among CD3^−^NK1.1^+^ cells at different gd (*n* ≥ 3). (*) Indicate statistical analysis of data from spleen, decidua, and uterus compared with liver at the same gd. (§) Indicate statistical analysis of data from decidua and pregnant uterus at different gd compared with virgin uterus. **(C,D)** CD3^−^NK1.1^+^ Eomes^+^ and Eomes^−^ cells isolated from decidua and uterus of Eomes-GFP mice at gd 5.5 were analyzed by flow cytometry for the indicated markers (black line), gray histograms correspond to unstained cells **(C)**, or splenic NK cells **(D)**; one representative experiment (*n* ≥ 3). **(E,F)** CD3^−^NK1.1^+^Eomes^+^ cells were analyzed for the expression of CD49a and CD49b. **(E)** One representative experiment (*n* = 6) at gd 5.5. **(F)** Mean ± SEM of percentage of the different subsets of CD3^−^NK1.1^+^Eomes^+^ cells at different gd in decidua and uterus (*n* ≥ 3). **(G,H)** Analysis of CD27 and CD11b expression in the CD3^−^NK1.1^+^Eomes^+^ cells subsets in pregnant uterus and decidua. **(G)** One representative experiment; **(H)** mean ± SEM of percentage of positive cells (*n* = 4).

Previous studies in mice indicated that during midgestation the majority of uterine CD3^−^NK1.1^+^ cells express high levels of Eomes (7, 33). Taking advantage of Eomes-GFP mice, we analyzed the expression of Eomes in dNK and uNK cells (identified as CD3^−^NK1.1^+^ cells) starting from the early phase of pregnancy (gd 5.5). The majority of dNK and uNK cells at gd 5.5 were Eomes^+^ and no differences were detected between pregnant and virgin uteri (Figure 5A). A small proportion of CD3^−^NK1.1^+^Eomes^−^ ILC1 was present in uterus and decidua at gd 5.5 and increased in percentages during pregnancy (Figure 5B). Both Eomes^+^ and Eomes^−^ cells were T-bet positive (not shown). Uterine and decidua NK1.1^+^Eomes^−^ cells expressed markers of tissue retention (CD49a, CD160, CD9, and CD69) and TRAIL, while they were negative for β_7_ integrin (Figures 5C,D). Notably, u- and d-Eomes^+^ cells displayed a bimodal expression of CD49a and β7 integrin, while they homogeneously expressed CD160, CD9, CD69, and TRAIL (Figures 5C,D).

**Figure 6:**
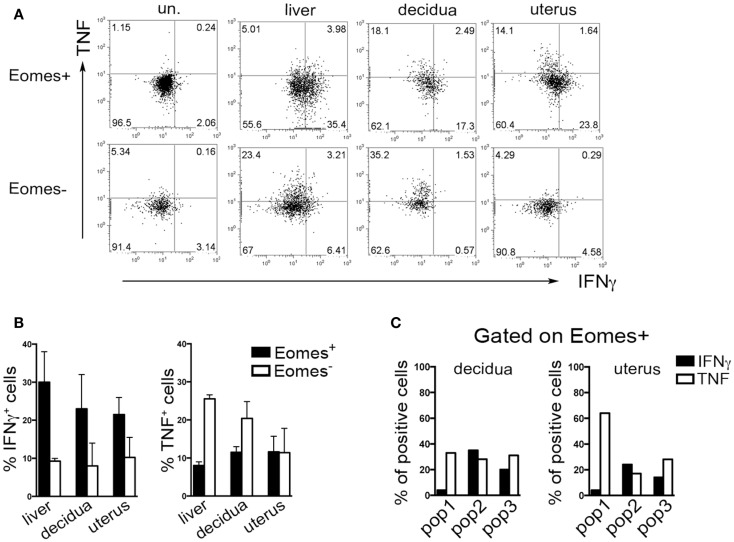
**Cytokines production by murine Eomes^+^ and Eomes^−^ cells**. **(A–C)** Decidua and uterus (CD3^−^NK1.1^+^) Eomes^+^ and Eomes^−^ cells were sorted from Eomes-GFP mice, stimulated with P + I for 18 h and analyzed for intracellular cytokine expression. **(A)** One representative experiment; **(B)** mean ± SEM of cytokine positive cells (*n* = 3); **(C)** percentages of IFNγ^+^ and TNF^+^ cells in the different Eomes^+^ cell subsets (see Figure 5F). Data derived from a pool of 10 mice.

Typically, CD49a identifies liver Eomes^−^ cells, while Eomes^+^ cNK cells are CD49a^−^CD49b^+^. Strikingly, the simultaneous analysis of Eomes, CD49a, and CD49b allowed the identification of three subsets of NK cells in uterus and decidua: Eomes^+^CD49a^+^CD49b^−^ (population, pop. 1), Eomes^+^CD49a^+^CD49b^+^ (pop. 2), and Eomes^+^CD49a^−^CD49b^+^ cells (pop. 3) (Figures 5E,F). The latter population (pop. 3) corresponds to cNK cells, while the other two subsets are only present in uterus and decidua. Notably, in decidua the percentages of Eomes^+^CD49a^+^CD49b^−^ (pop. 1) cells decreased during pregnancy, while cNK cells progressively increased (Figure 5F). Of note, Eomes^+^CD49a^−^CD49b^+^ (pop.3) cells are enriched in mature CD11b^high^CD27^low^ cells (24), while Eomes^+^CD49a^+^CD49b^−^ (pop. 1) and Eomes^+^CD49a^+^CD49b^+^ (pop. 2) contain higher percentages of cells displaying an immature phenotype (CD11b^low/high^CD27^high^), both in decidua and uterus (Figures 5G,H). In line with previous results (6), Eomes^+^ cells produced higher IFNγ and lower TNF than Eomes^−^ cells (Figures 6A,B). In decidua and uterus, Eomes^+^CD49a^+^CD49b^−^ (pop. 1) cells mainly produced TNF, while the two subsets of CD49b^+^ (pop. 2 and 3) cells expressed both TNF and IFNγ Figure 6C). Therefore, murine decidua and uterus contain different subsets of group 1 ILCs, including ILC1 (Eomes^−^CD49a^+^CD49b^−^IFNγ^low^TNF^high^), cNK (Eomes^+^CD49a^−^CD49b^+^IFNγ^high^TNF^low^), and two novel subsets of NK cells (Eomes^+^CD49a^+^CD49b^−^IFNγ^+^TNF^+^ and Eomes^+^CD49a^+^CD49b^+^IFNγ^+^TNF^+^) characterized by phenotypic and functional features shared by cNK cells and the formerly described trNK cells.

## Discussion

In the present study, we show that both human and murine uterine microenvironments are enriched in Eomes^+^ ILCs, i.e., NK cells. In particular, human endometrium and decidua NK cells include two subsets of CD103^+^ cells that could be further dissected on the basis of NKp44 expression. We found that decidual CD103^+^ NK cells produce higher amounts of IFNγ than CD103^−^ cells and express markers of tissue residency, that were maintained in culture with TGFβ. In murine uterus and decidua, Eomes^+^ cells are heterogeneous and, besides cNK cells, contain two peculiar subsets dissected by the expression of CD49a and CD49b. In particular, Eomes^+^CD49a^+^CD49b^–^ cells are mainly present during the early phase of pregnancy (gd 5.5) and characterized by TNF production.

Human NK cells represent the most abundant lymphoid population in decidual tissue during the first trimester of pregnancy. This, together with their peculiar phenotypic and functional features, raised many questions regarding their origin. Here, we show that dNK cells express markers suggestive of TGFβ imprinting, such as CD103 and CD9. Eomes^+^CD103^+^CD9^+^ NK cells are already detectable in the endometrium, thus, suggesting that the presence/recruitment of these ILC subsets does not depend on the pregnancy status. Stromal cells present in endometrium and decidua release TGFβ able to induce or maintain expression of these markers on NK cells and on dCD34-derived NK cells. These data suggest that both PB NK cells and dCD34^+^ precursors may be influenced by decidual microenvironment. Whether a similar TGFβ-dependent mechanism occurs also in mice remains to be determined. A population of CD103^+^ cells was described also in the human intestinal epithelia (8). Intestinal CD103^+^ cells homogeneously express NKp44 and are IFNγ producers. Conversely, most endometrial and decidual CD103^+^ cells do not co-express NKp44 and produce low levels of IFNγ as compared to PB NK cells. Nevertheless, among dNK cells, CD103^+^ cells represent the major source of IFNγ.

A recent report identified in mice Eomes^+^CD49a^+^ cells in the virgin uterus (7). Here, we show that these cells are also present in pregnant uterus and decidua and that CD49b expression allows the further identification of two subsets of Eomes^+^CD49a^+^ cells only detectable in these tissues. Eomes^+^CD49a^−^CD49b^+^ cNK cells, which are enriched in IFNγ producing cells, are the predominant Eomes^+^ subset during midgestation (gd 10.5) when they might contribute to spiral artery modification (34). On the other hand, Eomes^+^CD49a^+^CD49b^−^ cells mainly produce TNF, are abundant at gd 5.5 and subsequently decrease. A minor Eomes^−^CD49a^+^ cell population able to produce TNF is also present in decidua and uterus and increases during pregnancy. Thus, a source of TNF is constantly present during early and midgestation.

Studies aiming to characterize ILC subsets in several organs highlighted the complexity of this cell family and suggested that ILCs may display tissue-specific features and developmental requirements. In liver, it has been shown that T-bet^+^Eomes^+^ cNK and T-bet^+^Eomes^−^ ILC1 differentiate from precursors of medullary and peripheral origin, respectively (6). Our present data indicate that the majority of uNK and dNK cells express Eomes. They might derive from accumulation of circulating Eomes^+^ NK cells. However, in a previous study, we showed that only a minor fraction of dNK cells derives from the migration of splenic NK cells (21). Conversely, we showed that Lin^−^CD122^+^ NK precursors are present in murine decidua and uterus and give rise to immature NK cells that undergo differentiation during pregnancy (21). Thus, it is likely that Eomes^+^ NK cells may derive from *in situ* differentiation of precursors of medullary origin. In accordance with this hypothesis, hematopoietic precursors and immature NK cells capable of differentiating toward dNK cells are present also in human decidua and endometrium, supporting an *in situ* development of dNK cells (35, 36).

It is of note that also salivary gland NK cells, although expressing Eomes, display phenotypic features of trNK cells described in liver (13). Salivary gland Eomes^+^ NK cells are independent of *Nfil3* for their development, while uterine Eomes^+^ NK cells are reduced in *Nfil3*^−/−^ mice (13). Moreover, Colucci and co-workers (under review) show that *Nfil3* is required for the expansion of Eomes^+^CD49a^+^ NK cells during pregnancy. This, together with our present findings, suggests that peripheral tissues different from liver can sustain the differentiation of peculiar Eomes^+^ NK cells. The developmental relationship among the three subsets of Eomes^+^ NK cells identified here is unknown. NK cell maturation is a four-stage developmental program that starts at a CD11b^low^CD27^low^ stage and leads them through the following stages: CD11b^low^CD27^high^ → CD11b^high^CD27^high^ → CD11b^high^CD27^low^ (24). Moreover, CD49b is acquired during NK cell differentiation (37). Here, we show that, among Eomes^+^ cells, CD49a^+^CD49b^−^ cells decrease in percentages during pregnancy, while CD49a^−^CD49b^+^ cells increase. Moreover, CD49a^−^CD49b^+^ cells are enriched in mature CD11b^high^CD27^low^ cells, while CD49a^+^CD49b^−^ and CD49a^+^CD49b^+^ cells contain higher percentages of immature CD11b^low^CD27^high^ cells, both in uterus and decidua. It may be possible to speculate that the three subsets of Eomes^+^ NK cells represent developmentally related differentiation stages, although fate-mapping experiments will be needed to test this hypothesis.

Thus, our data indicate that the formerly indicated uNK and dNK actually represent heterogeneous group 1 ILC populations, including Eomes^−^ ILC1, Eomes^+^ cNK cells, and two novel subsets of Eomes^+^ NK cells, phenotypically and functionally distinct from trNK previously described in other tissues. It is conceivable that they may diverge from the Eomes^+^-developmental pathway under the influence of decidual microenvironment that may contribute to their differentiation. Further studies will be required for better understanding the role of the different ILC subsets in the establishment and maintenance of pregnancy.

## Author Contributions

EM, PV, and LC designed the study, performed experiments, analyzed data, and wrote the manuscript; DC performed experiments and analyzed data; FL and SM performed experiments; SF selected human samples and interpreted data; TW interpreted data and revised the manuscript; LM and MM supervised the study and wrote the manuscript.

## Conflict of Interest Statement

The authors declare that the research was conducted in the absence of any commercial or financial relationships that could be construed as a potential conflict of interest.
